# Supplementary material for: Factors shaping vaginal microbiota long-term community dynamics in young adult women
Source: Peer Community J. Author manuscript; Available in PMC 2025 Mar 17. (PMC7617500; doi:10.24072/pcjournal.527)
Supplement: Appendix [file EMS203670-supplement-Appendix.pdf]

Appendix A. Pairwise correlations between covariates

No strong correlations were observed among covariates, with the strongest correlation found between BMI and stress ($r = 0.41$; Fig. S1).

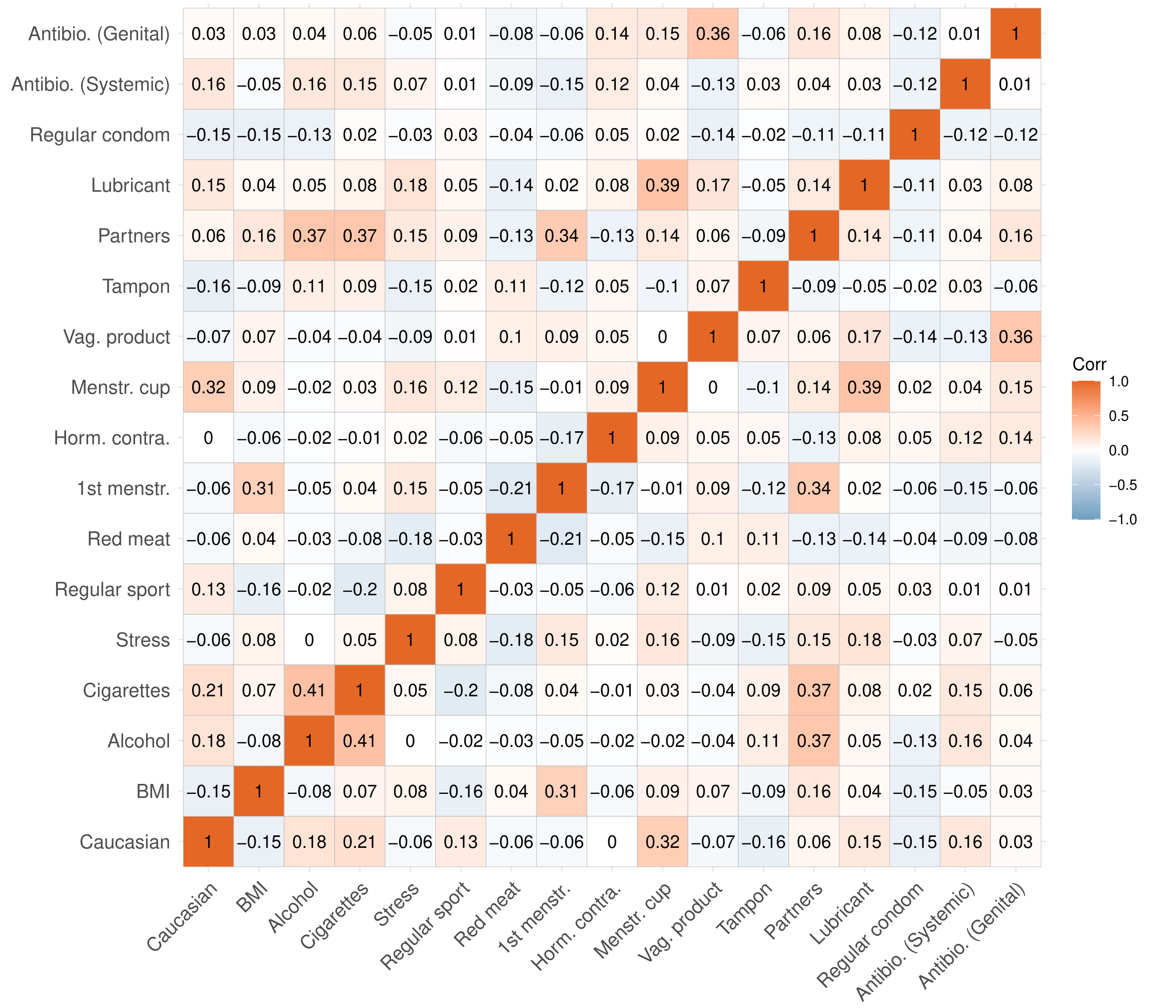

Figure S1 – Correlation between covariates. Pairwise Pearson's correlation coefficients between covariates. Parameter descriptions can be found in Materials and Methods.

Appendix B. Assessment of posterior accuracy, precision and prior contraction

We leveraged the properties of posterior distributions to identify potential model fitting problems that might manifest from our model assumptions. To examine the accuracy and precision of posterior distributions, we first generated simulated observations based on the estimated posterior mean parameters. We then refitted our model to the simulated observations (i.e., secondary fitting) to compute the posterior z-score for each parameter, which measures how closely the posterior recovers the parameters of the data generating process (Betancourt, 2020):

$$z = \frac{\mathbb{E}_{\text{sim}} - \mathbb{E}_{\text{post}}}{\sigma_{\text{sim}}},$$

where $\mathbb{E}_{\text{post}}$ denotes the posterior mean of the fit to the actual data that we consider the "true" parameter. $\mathbb{E}_{\text{sim}}$ and σ_{sim} denote the mean and standard deviation of the posterior distribution of the secondary fitting. The smaller the z-score, the closer the bulk of the posterior is to the

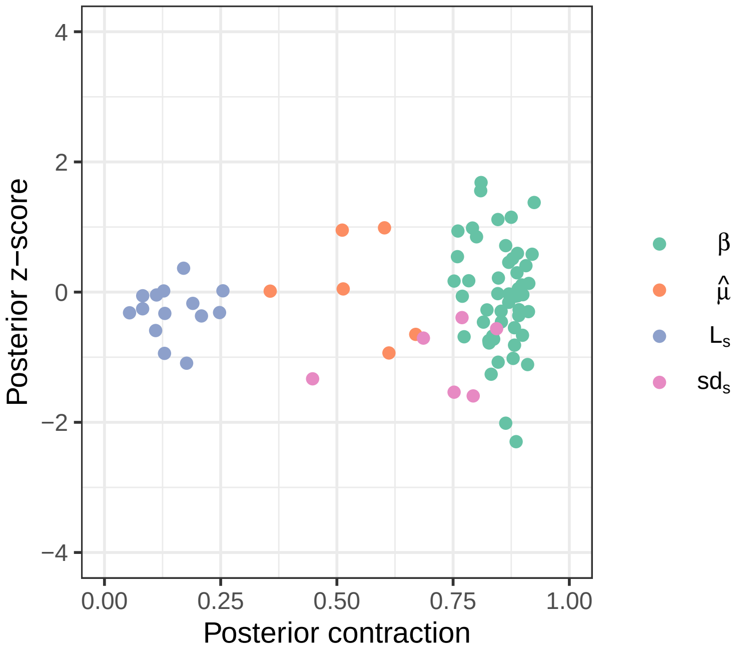

Figure S2 – Accuracy, precision and identifiability of estimated parameters. Posterior z-score (y-axis) measures how closely the posterior recovers the parameters of the true data-generating process and posterior contraction (x-axis) evaluates the influence of the likelihood function over the prior, respectively. Smaller absolute posterior z-scores indicate that the posterior accurately recovers the parameters of the data-generating process: the absolute value beyond three to four may indicate substantial bias (Betancourt, 2020). The posterior contraction values close to one indicate that data are much more informative than the prior. The estimated parameters are represented by a filled dot.

true parameter (Betancourt, 2020). In contrast, large z-values may be indicative of overfitting or poor prior specifications (Betancourt, 2020).

To examine the influence of the likelihood function in relation to prior information, we computed the posterior contraction, k:

$$k = 1 - \frac{\sigma^2_{\text{post}}}{\sigma^2_{\text{prior}}}$$

where σ^2_{post} and σ^2_{prior} correspond to the variance of posterior and prior distributions, respectively. The k values close to zero indicate that data contain little information (i.e., rendering priors strongly informative). Conversely, values close to 1 indicate that data are much more informative than the prior (Betancourt, 2020).

We found that most of our model parameters and hyperparameters — were estimated with accuracy, precision, and identifiability, with the absolute posterior z-scores below three (Fig. S2). The posterior distributions for covariate coefficients, β, contracted by 86 % on average, and at least 75 %, compared to the prior distribution, meaning that the covariate coefficients were well-identified from data (Fig. S2). Although we used generic priors recommended by Stan (Stan Development Team, 2024), the L_s parameters that define correlations among between-woman variation showed limited posterior contraction (i.e., $\leq\sim 0.25$), indicating that these parameters are poorly informed by data. As such, we refrain from making biological inferences about these correlations.

Appendix C. Predicted difference in community state type (CST) prevalence at various counterfactual scenarios.

Our counterfactual simulations predicted that alcohol consumption and the number of partners are factors that impact the population-level outcome in terms of the prevalence of different community state types. The full list of comparisons is available in Fig. S3.

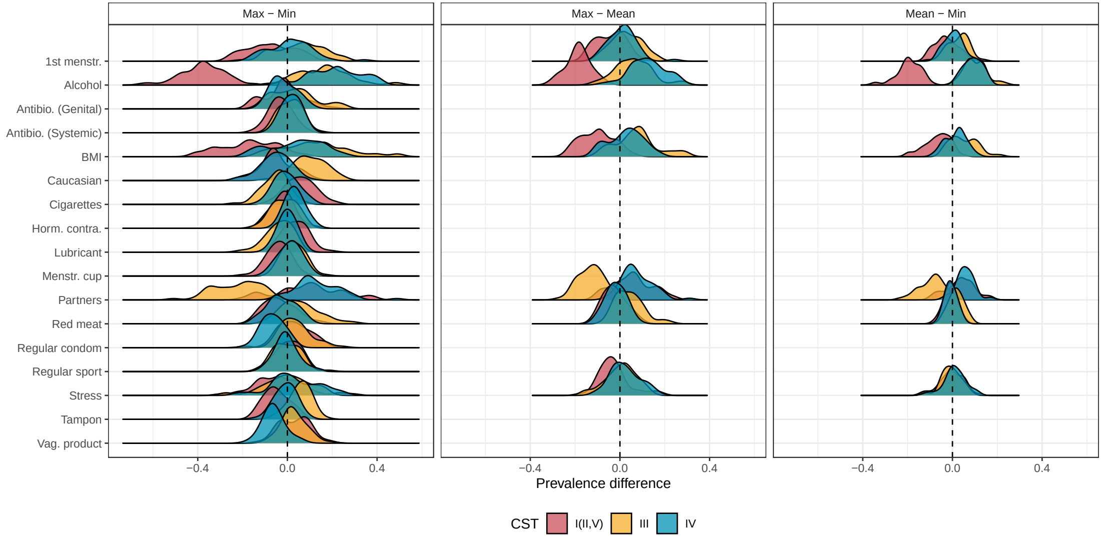

Figure S3 – Difference in community state type (CST) prevalence at predicted various counterfactual scenarios. The differences were calculated from posterior samples simulated at 0 and 1 for binary variables and at the population maximum and minimum values recorded by the PAPCLEAR for continuous variables (left panel). Additional differences were computed between the population maximum and mean (middle panel) and the population mean and minimum for continuous variables (right panel). Parameter descriptions can be found in Materials and Methods.
